# Coronavirus disease (COVID-19) and conjunctival sac swab findings

**DOI:** 10.1186/s12348-022-00285-3

**Published:** 2022-02-19

**Authors:** Pavol Vesely, Elena Novakova, Michal Stubna, Michal Trnka, Denisa Jurenova, Darina Lyskova, Robert Furda, Paulina Plesnikova, Vratko Himic, Alena Furdova

**Affiliations:** 1 Department of Ophthalmology, Faculty of Medicine, Comenius University, (Klinika oftalmológie LFUK a UNB), Hospital Ruzinov, Ružinovská 6, 826 06 Bratislava, Slovak Republic; 2 Department of Microbiology and Immunology, Jessenius Faculty of Medicine in Martin, Comenius University, Bratislava, Slovakia; 3 Department of Ophthalmology, Hospital Zilina, Zilina, Slovakia; 4 Department of Medical Physics, Biophysics, Informatics and Telemedicine, Faculty of Medicine, Comenius University, Bratislava, Slovakia; 5 Department of Information Systems, Faculty of Management, Comenius University in Bratislava, Bratislava, Slovakia; 6 Department of Physiology, Anatomy and Genetics, University of Oxford, Oxford, UK

**Keywords:** COVID-19, Lacrimal apparatus, Analysis, Polymerase chain reaction

## Abstract

**Background:**

The purpose of this article is to evaluate the positivity of conjunctival sac swab by PCR (Polymerase chain reaction) test in COronaVIrus Disease 19 (COVID-19) patients.

**Methods:**

Inclusion criteria of our study were COVID-19 patients hospitalized during March 2021 in inpatient wards at University Hospitals in towns Bratislava and Zilina, Slovakia. The conjunctival sac swabs collected by four ophthalmologists were stored for 24 h, then analyzed in the laboratory of the Department of Microbiology and Immunology, Jessenius Faculty of Medicine in Martin, Comenius University, Slovakia. The sampling apparatus, used for conjunctival sac swab, was the Dacron polyester swab.

**Results:**

We examined one group of 302 COVID-19 patients, 168 Male (56%) and 134 Female (44%). The patients’ mean age was 66.3 ± 13.66 years, ranging from 25 to 96 years, and the mean length of hospital stay in our patients with a nasopharyngeal positive PCR test was 7.33 ± 4.76, from 2 to 24 days.

The PCR tests from the conjunctival sac swabs were positive in 33 patients (11%), negative in 259 patients (86%), and ten patients (3%) were with the unclear result. In the group of 33 positive patients were 17 males with a mean age of 74.6 ± 13.59 years and 16 females with a mean age of 70.63 ± 14.17 years.

The cycle threshold (C_T_) values differed significantly between conjunctival sac swabs from the nasopharynx and the conjunctiva. Medians of the values were 25.1 (14.1, 32.1) and 31.5 (22.6, 36.6) (*P* <  0.001), respectively.

**Conclusion:**

This study affirmed that in COVID-19 patients the SARS-CoV-2 was detectable with PCR test in conjunctival sac swab, but the positivity rate was only about one to ten cases (11%).

## Introduction

COronaVIrus Disease 19 (COVID-19) is caused by the severe acute respiratory syndrome coronavirus-2 (SARS-CoV-2). In the last 2 years, the disease has quickly become a worldwide health problem since its first detection in December 2019 in China [1].

Respiratory symptoms and myalgias are the fundamental clinical picture of COVID-19, but in addition, the conjunctivitis has been recognized as an additional clinical manifestation associated with SARS-CoV-2 infection [2, 3]. In some studies in the past 2 years, the presence of SARS-CoV-2 Ribonucleic acid (RNA) in conjunctival sac and tears has also been reported in COVID-19 patients [4–7].

The primary routes of transmission of SARS-CoV-2 infection are the most frequent via respiratory droplets. The SARS-CoV-2 RNA has been also detected in tears and conjunctival sac from COVID-19 patients. Clinical symptoms, like conjunctivitis, can occur alongside the other COVID-19 symptoms, or it may be the only sign of the disease [7, 8]. The purpose of this article is to evaluate the positivity of conjunctival sac swab by PCR (Polymerase chain reaction) test in COVID-19 patients.

## Materials and methods

We investigated 302 patients with the clinical diagnosis of COVID-19 and the combination of clinical symptoms that included positive nasopharyngeal RT-qPCR test. The clinical symptoms have been confirmed from the patient records, by including the ocular symptoms.

Inclusion criteria of our study were the COVID-19 patients hospitalized during March 2021 in inpatient wards at University Hospitals in towns Bratislava and Zilina, Slovakia. The conjunctival sac swab was analyzed by PCR test. The sampling apparatus was the Dacron polyester swab with the NADAL® COVID-19 IgG/IgM (Nal von Minden GmbH, Moers, Germany) sample. The conjunctival sac swabs were collected by four ophthalmologists and stored for 24 h. On the next day, the analysis was performed in a laboratory of the Department of Microbiology and Immunology, Jessenius Faculty of Medicine in Martin, Comenius University, Slovakia.

### RNA isolation

RNA isolation from the conjunctival sac swabs with the concentrated virus was performed using a Quick-RNA™ Viral 96 kit (Zymo Research, Waltham, MA, USA, cat#R1041) isolation kit, as recommended by the manufacturer, with a slight modification: 200 μl of DNA/RNA Shield TM was added to 200 μl of transport medium containing the conjunctival sac swab. These conjunctival sac swabs were then mixed with viral RNA buffer and transferred to a Zymo-Spin™ I-96 Plate. After washing and centrifugation, viral RNA was transferred with 25 μl DNase/RNae-free water to the elution plate and then used in an RT-qPCR test.

### RT-qPCR test

We used the Multiplexed rTest set (rTEST COVID19/FLU qPCR Kit) from MultiplexDX™ International, according to the manufacturer’s requirements. This kit is a re-designed version of the WHO (World Health Organization) recommended Charité, Berlin protocol, along with a newly designed differential test that can distinguish between SARS-CoV-2, IAV (Influenza A Virus) and IBV (Influenza B Virus). RT-qPCR was performed on qTOWER^3^ (Analytic Jena GmbH, Jena, Germany) with standard Thermal Cyclers. The viral load in the initial conjunctival sac swab was estimated by specifying the threshold cycle (C_T_) of the SARS-CoV-2. The results were analyzed using qPCRsoft (real-time PCR control and evaluation software) with a constant threshold calculation for the determination of C_T_ values.

### Statistical analysis

Continuous variables were expressed as means ± standard deviations, and categorical variables as frequencies and percentages. Comparisons of means between groups were performed using a paired t-test for normally distributed continuous variables. All tests were carried out at the significance level of α = 0.001. (The two-tailed *p*-value of < 0.001 was considered statistically significant.) Data analysis was conducted by the statistical software IBM SPSS version 27 (IBM SPSS Inc., Armonk, NY, USA).

## Results

In 302 patients, we examined, the median age was 66.3 ± 13.66 years (Table 1), ranging from 25 to 96. From the group, 168 patients were males with the median equal to 63.5 years and the mean 64.95 ± 13.88 years, and 134 female patients with the median equal to 69.0 years and the mean 68.01 ± 13.23 years. To specify the status of the patients in inpatient care, there 262 patients (93%) were on nonmechanically ventilated oxygen therapy and 13 patients (4.3%) were on mechanically ventilated oxygen therapy, 24 h a day.

**Table 1:** The group of all COVID-19 patients and its subgroup with positive PCR test in conjunctival sac swab

Gender	No	Age ^**a**^	Nonmechanically ventilated patients ^**b**^	Mechanically ventilated patients ^**b**^	Positive conjunctival sac swab	Ocular symptoms ^**b**^
Female	134	68.01 ± 13.23	116 [15]	8 [1]	16	20 [1]
Male	168	64.95 ± 13.88	146 [16]	5 [1]	17	32 [3]
Overall	302	66.30 ± 13.66	262 [31]	13 [2]	33	52 [4]

^a^Mean (in years) and Standard Error of Mean (SEM) were calculated, and were showed as Mean ± SEM

^b^Patients [Patients with positive PCR test in conjunctival sac swab]

The PCR tests from the conjunctival sac swabs were positive in 33 patients (11%), negative in 259 patients (86%), and ten patients (3%) were with the unclear result. In the group of 33 positive patients were 17 males with a mean age of 74.6 ± 13.59 years and 16 females with a mean age of 70.63 ± 14.17 years. To specify the status of the patients in inpatient care, there 31 patients were on nonmechanically ventilated oxygen therapy and two patients were on mechanically ventilated oxygen therapy, 24 h a day.

Ocular symptoms (itching and red eye) were documented in 52 patients (17%) – for the details see the Table 1 and the Table 2.

**Table 2:** The comparison of the number of all COVID-19 patients with the subset of those who were with positive PCR test in conjunctival sac swab stratified by age and gender

Age group	Female ^**a**^	Male ^**a**^	Nonmechanically ventilated patients ^**b**^	Mechanically ventilated patients ^**b**^	Ocular symptoms ^**b**^
20	1 [0]	1 [0]	1 [1]	0 [0]	0 [0]
30	3 [0]	5 [0]	2 [5]	0 [0]	0 [0]
40	11 [2]	18 [1]	7 [14]	2 [0]	1 [6]
50	16 [2]	38 [1]	11 [32]	3 [2]	4 [6]
60	44 [3]	45 [4]	42 [33]	1 [3]	8 [10]
70	35 [5]	35 [5]	30 [35]	2 [0]	5 [5]
80	22 [3]	21 [3]	21 [21]	0 [0]	2 [5]
90	2 [1]	5 [3]	2 [5]	0 [0]	0 [0]

^a^Patients [Patients with positive PCR test in conjunctival sac swab]

^b^Female [Male]

**Fig. 1:**
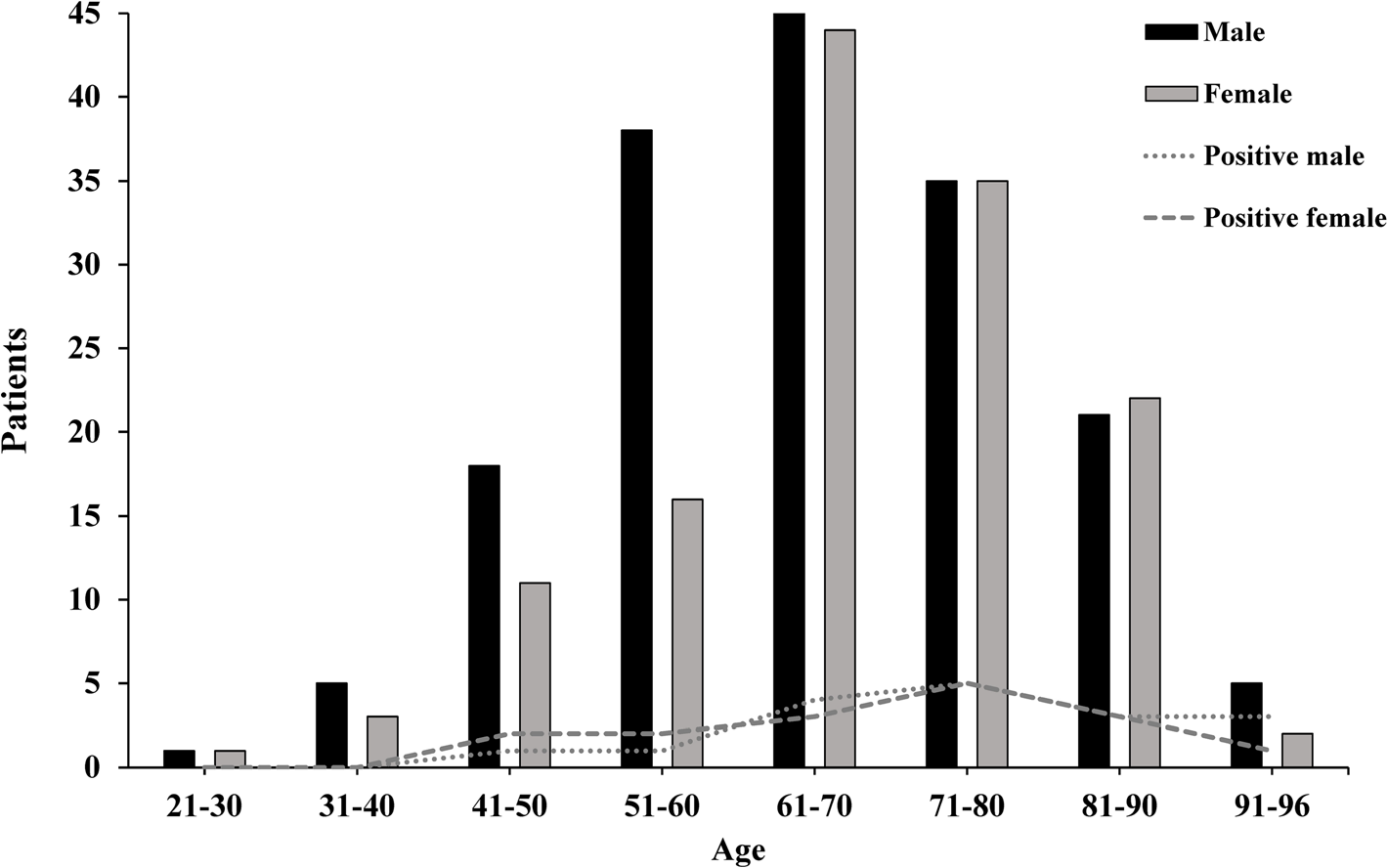
The numeric comparison of all COVID-19 patients, by age and gender, and the subgroup with positive PCR test in conjunctival sac swab

In Fig. 1, we compare all COVID-19 positive patients, grouped by age and gender, and its subgroup with positive PCR test in conjunctival sac swab.

In Table 2 we document male and female patients in the age groups at ten-years intervals, where, for example, the 20 years age group represents the interval from 20 to 29 years.

The cycle threshold (C_T_) values varied significantly between conjunctival sac swabs from the nasopharynx and conjunctiva, with a median value of 25.1 (14.1, 32.1) and 31.5 (22.6, 36.6) (*P* <  0.001), respectively (Table 3). The cycle threshold (C_T_) values between nasopharynx and conjunctiva were different for all patients when stratified by age and gender (Table 4).

**Table 3:** The cycle threshold (C_T_) values from patients with positive PCR test in conjunctival sac swab

Patients	No	C_**T**_ value in nasopharynx ^**a**^	C_**T**_ value in conjunctiva ^**a**^	*p*
Female	16	23.58 ± 5.53	31.44 ± 3.92	< 0,001
Male	17	23.38 ± 4.73	29.91 ± 3.31	< 0,001
Overall	33	23.48 ± 5.06	30.65 ± 3.64	< 0,001

^a^Mean and Standard Error of Mean (SEM) were calculated, and were showed as Mean ± SEM

**Table 4:** The cycle threshold (C_T_) values from patients with positive PCR test in conjunctival sac swab stratified by age and gender

Age group (years)	Female			Male		
	No	C_**T**_ value in nasopharynx ^**a**^	C_**T**_ value in conjunctiva ^**a**^	No	C_**T**_ value in nasopharynx ^**a**^	C_**T**_ value in conjunctiva ^**a**^
40	2	22.56 ± 5.74	32.49 ± 0.66	1	29.80 ± 0.00	33.54 ± 0.00
50	2	20.45 ± 7.85	34.61 ± 0.04	1	25.00 ± 0.00	30.45 ± 0.00
60	3	29.57 ± 2.48	31.92 ± 2.97	4	23.65 ± 6.24	30.03 ± 4.14
70	5	23.63 ± 5.51	30.90 ± 4.92	5	22.28 ± 5.16	28.60 ± 3.68
80	3	23.47 ± 2.24	31.99 ± 2.35	3	24.03 ± 3.68	30.45 ± 3.19
90	1	14.00 ± 0.00	22.56 ± 0.00	3	21.51 ± 4.78	29.99 ± 3.62

^a^Mean and Standard Error of Mean (SEM) were calculated, and were showed as Mean ± SEM

## Discussion

The similar study of Kumar et al. [7] shows that SARS-CoV-2 can be detected in conjunctival swabs in patients with confirmed COVID-19 disease. In their results, the positivity rate of detecting SARS-CoV-2 in conjunctival swabs was low, there only one patient (2.23%) out of the group of 45 patients was positive, none of the patients had any ocular symptoms, and the patient’s cycle threshold value in conjunctival swab was 33, for the real-time RT-PCR SARS-CoV-2. In our study, we investigated more than six times more patients, the positivity in conjunctival sac swab was more than four times higher (11%), we found ocular symptoms (itching and red eye) in 52 patients (17%), and patients, who were also divided into age groups at ten-years intervals, the cycle threshold values were significantly different between the conjunctival sac swabs from the nasopharynx and conjunctiva, with median value of 25.1 and 31.5 (*P* < 0.001), respectively. The C_T_ values between nasopharynx and conjunctiva were different for all patients when stratified by age and gender in our group.

SARS-CoV-2 obtains passage via angiotensin-converting enzyme 2 (ACE-2) receptor, which may express in various tissues, including the conjunctiva [9]. During the SARS-related coronavirus flare-up in 2003, an investigation showed that medical personnel experienced a more significant danger of SARS contamination when there was an unprotected eye-to-eye connection with discharges. There is an increasing trend in reports indicating that some COVID-19 pneumonia cases began with conjunctivitis as an underlying feature after contact with infected individuals. Detection of viral RNA by RT-qPCR can be valuable in the early detection of COVID-19 and in taking appropriate isolation measures. Then, determining whether SARS-CoV-2 is transmissible by contacting the conjunctival sac importantly considers that warrants investigation [10–12].

SARS-CoV-2 RNA has recognition in tears of patients with COVID-19 both with and without conjunctivitis. Be that as it may, gathering tears and visual discharges for SARS-CoV-2 detection appears to have limited benefit [13, 14].

During the COVID-19 pandemic, more authors published reports highlighting various ocular signs of the infection. One meta-analysis discovered that the general preponderance of ocular symptoms was around 11% [15]. The most frequent eye symptoms in COVID-19 patients were ocular pain, redness, and follicular conjunctivitis. Based on the current studies, SARS-CoV-2 we can verify by swab in conjunctival secretion when analyzing it with RT-PCR. In our approach, we found 33 positive patients (11%).

Only a few authors have suggested utilizing eye protection to evade the projected transmission of COVID-19. These proposals incorporate techniques to forestall transmission of disease among ophthalmologists and optometrists. They are produced from ocular systems, for example, during cataract surgery and non-contact tonometry [16–21].

The data of one study with meta-analysis uncovered the extent of different visual features, for example, visual acuity decline, redness, release, and follicular conjunctivitis. Another study announced similar ocular symptoms. In addition, these studies depended on itemized and comprehensive surveys and patient meetings, which occurred a few days after the patients left the medical inpatient clinic [21, 22]. Subsequently, the information from the examinations could be subject to the bias of review inclination. Likewise, it isn’t sure whether these visual features preceded COVID-19 or happened as a consequence of that. For example, symptoms like dry eyes, tingling, and foreign body sensation might be profoundly normal in every COVID-19 patient. Also, certain studies have included healthcare workers who might be more sharpened on detailing different indications. Then again, in dangerous circumstances, the more extreme clinical indicators may overshadow ophthalmic symptoms, which may go unnoticed. In addition, the studies raised the worry that COVID-19 can have the ocular manifestations (explicitly follicular conjunctivitis) as first and in various cases the single symptom of the disease. Pooled information from three investigations in the meta-examination uncovered that the visual side effects might be the main look in roughly 2.2% of patients. It is essential to be aware of such cases and keep clinical suspicion of COVID-19 in such patients [21, 23–26].

In one systematic review, the authors compared over 200 ophthalmology-focused scientific articles with the COVID-19 pandemic they published during the last months, reporting the presence of conjunctivitis and other varied ocular manifestations [27]. Our results contributed to this rapidly growing topic.

## Conclusion

In summary, manifestations like redness, pain, and conjunctivitis may occur in COVID-19 patients. This study confirmed that in COVID-19 patients, the SARS-CoV-2 can also be detected in conjunctival sac swab by PCR test. The positivity rate is only about one to ten cases (11%). However, the detection speed of viral RNA from conjunctival sac swab and tear fluid with the PCR test is poor.

## Data Availability

The datasets used and/or analyzed during the current study are available from the corresponding author on reasonable request.
